# The invasive pest *Drosophila suzukii* uses trans-generational medication to resist parasitoid attack

**DOI:** 10.1038/srep43696

**Published:** 2017-03-13

**Authors:** M. Poyet, P. Eslin, O. Chabrerie, S. M. Prud’homme, E. Desouhant, P. Gibert

**Affiliations:** 1 Univ. Lyon, Université Claude Bernard Lyon 1, CNRS, Laboratoire de Biométrie et Biologie Evolutive, F-69100, Villeurbanne, France; 2 Unité Ecologie et Dynamique des Systèmes Anthropisés (FRE-CNRS 3498), Université de Picardie Jules Verne, Amiens, France

## Abstract

Animal medication is a behavioral strategy to resist enemies based on the use of substances from the environment. While it has been observed in several animals, whether invasive species can use medication to resist new enemies during its expansion is unknown. Here, we show that the worldwide invasive pest *Drosophila suzukii* performs trans-generational prophylactic medication by adapting its oviposition behavior in the presence of enemies. We find that flies preferentially lay their eggs on media containing atropine – an entomotoxic alkaloid – in the presence of parasitoids. We further show that flies developing on atropine more efficiently resist parasitization by parasitoids. Finally, we find that developing in hosts reared on atropine strongly impacts the life-history traits of parasitoids. This protective behavior is reported for the first time in a pest and invasive species, and suggests that animal medication may be an important driver of population dynamics during invasions.

Trapped between the bottom-up control of food quality and the top-down control of natural enemies, phytophagous insects need to develop a wide variety of responses to survive. Among these responses, there is growing attention on behavioral resistance strategies that make use of environmental molecules, commonly referred to as animal medication^1^,^2^. Animal medication is a behavioral strategy used by some species to clear a natural enemy or to reduce its symptoms by taking advantage of molecules produced by some plant species^1^,^2^. This behavioral strategy is not necessarily employed by an individual for its own protection (self-medication); it can also be effective to protect its offspring (trans-generational medication)^3^,^4^,^5^. For example, *Drosophila melanogaster* females preferentially choose to lay their eggs in ethanol-laden food after visual detection of natural enemies, parasitoid wasps^6^. The survival rate of parasitized larvae that developed on this ethanol substrate, present in fermenting fruits in nature, is increased without the need for the fly to develop immune responses^7^.

Considering its potential impact on multi-trophic systems, animal medication behavior should be an important driver of interspecific interactions and population dynamics. In particular, during their introduction into new areas, exotic species have to face a new range of natural enemies that can hamper their spread^8^,^9^. All potential resistance mechanisms against these new enemies can contribute to their invasion success into native communities. For instance, several hypotheses (‘Enemy Release Hypothesis’^10^, ‘Evolution of Increased Competitive Ability’^11^) highlight how exotic species can benefit from a decrease in regulation by natural enemies contributing to the success of a biological invasion. Medication behaviors could be one of these resistance strategies.

Here, we tested the trans-generational medication strategy in the case of an invasive species, the spotted-wing drosophila, *Drosophila suzukii*. This species is a successful invasive fruit fly which originated in Asia and then spread through Europe and the United States starting in 2008^12^,^13^,^14^. *D. suzukii* is highly polyphagous, meaning it is able to feed on a wide range of wild and ornamental plants^15^,^16^. Intriguingly, *D. suzukii* was previously shown to exhibit a high oviposition and development rate in lab conditions on the fruits of *Atropa belladonna* (Solanacea)^15^, a plant species with high concentrations of entomotoxic alkaloids, mostly atropine^17^,^18^. It develops also on *Atropa belladonna* fruits *in natura* in the region of Amiens, France, since 2011 (O. Chabrerie, personal observation).

We hypothesized that *D. suzukii* could use the entomotoxic property of atropine to medicate against its principal enemies in Europe (*i.e.* parasitoids parasitizing flies in development). Immature flies grow on the substrate in which their mother oviposits. Consequently we postulated that to benefit from the properties of atropine against parasitoids, growing individuals would have to be laid by females on atropine-containing fruits. We conducted behavioral experiments to verify whether the necessary conditions of existence of trans-generational medication (defined in refs 5,6 and 19) occur in the biological model under study. We predicted that: i) *D. suzukii* immature developed on atropine-laden food remain a potential target for parasitization; ii) in the case of parasitic presence, *D. suzukii* females choose to lay eggs on atropine laden-food; iii) the fitness of parasitized *D. suzukii* developed on atropine-laden food is increased in comparison with individuals that grew on substrate without atropine, while iv) the fitness of parasitoids emerging from larvae that grew on atropine-containing substrates is decreased.

## Results

### Development in atropine media does not prevent *Drosophila suzukii* from parasitization

We showed that parasitoid females did not lay different numbers of eggs on drosophila immatures grown either on regular medium or on atropine medium. *Trichopria.* cf. *drosophilae* females laid 46% of their eggs on *D. suzukii* pupae reared on regular medium and 54% on *D. suzukii* pupae reared on atropine medium (binomial test; p-value = 0.48). Similarly, *Asobara japonica* females laid 49% of their eggs on *D. suzukii* pupae reared on regular medium and 51% on *D. suzukii* pupae reared on atropine medium (binomial test; p-value = 0.92). *D. suzukii* immatures fed on atropine medium therefore remained potential targets to parasitoid oviposition. This result indicated that laying eggs on atropine-containing substrates and using atropine alkaloid as a repellent for enemies was not a way for *D. suzukii* to avoid parasitization.

### *Drosophila suzukii* females modify their oviposition strategy when in the presence of parasitoids

**Figure 1:**
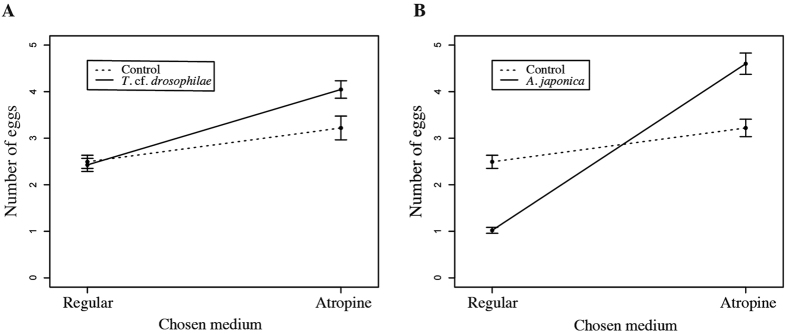
Number of eggs laid by *D. suzukii* on regular or atropine medium in the absence (dotted lines) or presence of parasitoids (solid lines). (**A**) In the absence or presence of *T.* cf. *drosophilae* (n = 200 experimental replicates). (**B**) In the absence or presence of *A. japonica* (n = 200 experimental replicates). Bars represent Standard Errors of the mean.

*D. suzukii* females preferred to lay eggs on an atropine-containing medium in the presence of both *T.* cf. *drosophilae* (interaction *choice* × *parasito*: χ^2^_1_ = 29.92, p-value = 0.016; Fig. 1A) or *A. japonica* (interaction *choice* × *parasito*: χ^2^_1_ = 304.99, p-value < 0.0001 Fig. 1B). Plus, irrespective of the presence of *A. japonica* or *T.* cf. *drosophilae*, we did not observe a significant effect of the original developmental media of the fly (*devR*) on its subsequent oviposition strategy. Finally, the total number of eggs laid by *D. suzukii* was higher in the presence of *T.* cf. *drosophilae* (due to additional eggs in atropine-laden medium, Fig. 1A) than in control tests (χ^2^_1_ = 21.66, p-value = 0.041), suggesting a stimulation of its oviposition frequency.

### Atropine in drosophila developmental medium modified the outcome of drosophila-parasitoid interaction

**Figure 2:**
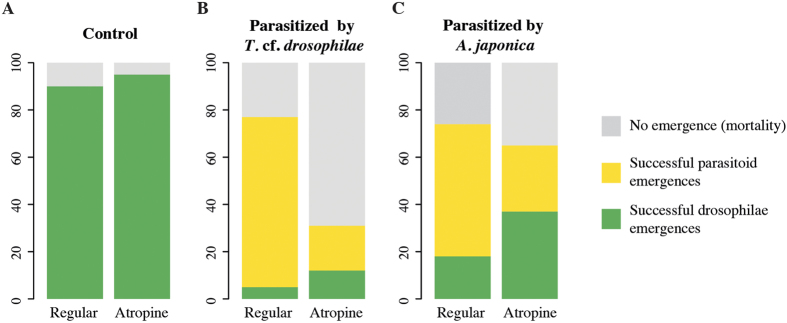
The effect of atropine on drosophila-parasitoid interaction. We recorded emergences (**A**) in control conditions, without parasitic pressure; (**B**) in case of parasitization by *T.* cf. *drosophilae*; (**C**) in case of parasitization by *A. japonica*. Light gray boxes: Mortality Rate of the fly-parasitoid pairs; Yellow boxes: Successful parasitoid emergence; Green boxes: Successful drosophila emergences (n = 100 experimental replicates for each modality).

In control conditions, without parasitoids, the number of *D. suzukii* individuals that emerged on regular or atropine medium was not significantly different (χ^2^_1_ = 0.135, p-value = 0,713; Fig. 2A). However, the developmental time of *D. suzukii* individuals that emerged on atropine medium was significantly longer than the developmental time of individuals that emerged on regular medium (W = 2622.5, p-value < 0.0001, size effect (Cohen’s d) = 0.67; data in Annex 2).

After a parasitization by *T.* cf. *drosophilae* (Fig. 2B), the number of emerging *D. suzukii* was not statistically different between the two media in which they developed (interaction *devD* × *parasito*: χ^2^_1_ = 2.39, p-value = 0.12). We only observed a tendency for an increased resistance (emergence rate rising from 5% (n = 100) to 12% (n = 100)) in drosophila reared on atropine medium. In the presence of *A. japonica*, the effect of the interaction between developmental medium and parasitoid on emerging flies was significant: the number of host larvae that resisted parasitization was greater when the drosophila developed on atropine medium (interaction *devD* × *parasito*: χ^2^_1_ = 4.43, *p-value* = 0.03). The percentage of emerging *D. suzukii* increased from 18% (n = 100) to 37% (n = 100) (Fig. 2C).

In contrast, we observed a negative effect of atropine on the success of the parasitoid development of both *T.* cf. *drosophilae* and *A. japonica* (Fig. 2B and C). The number of emerging *T.* cf. *drosophilae* drastically decreased from 73% (n = 100) to 20% (n = 100) (χ^2^_1_ = 32.9, *p-value* < 0.0001) when emerging from hosts reared on atropine medium (Fig. 2B). Similarly, the emergence rate of *A. japonica* decreased from 58% (n = 100) to 31% (n = 100) (χ^2^_1_ = 9.51, *p-value* = 0.0020; Fig. 2C) when emerging from drosophila reared on atropine medium.

Finally, we showed that the number of dead pairs of drosophila-parasitoid was much higher on atropine medium than on regular medium. For the pair *D. suzukii* - *T*. cf. *drosophilae*, the mortality rate was significantly increased from 22% (n = 100) to 67% (n = 100) (χ^2^_1_ = 24.06, *p-value* < 0.0001) when *D. suzukii* was originally reared on atropine medium. The same but a non-significant increasing tendency from 22% (n = 100) to 43% (n = 100) was observed after parasitization by *A. japonica* (χ^2^_1_ = 1.33, *p-value* = 0.2483).

### Atropine in drosophila developmental medium had a strong negative effect on parasitoid life-history traits

Parasitoids that emerged out of *D. suzukii* that fed on atropine medium were significantly smaller than parasitoids that emerged out of drosophila fed on regular medium. Tibia sizes decreased by 12%, from 57.32 μm (SE = 1.48) to 44.85 μm (SE = 1.86) (χ^2^_1_ = 12.18, *p-value* = 0.0004) for *T. cf. drosophila,* and by 22%, from 116.23 μm (SE = 1.57) to 91.79 μm (SE = 2.09) (χ^2^_1_ = 47.59, *p-value* < 0.0001) for *A. japonica.*

**Figure 3:**
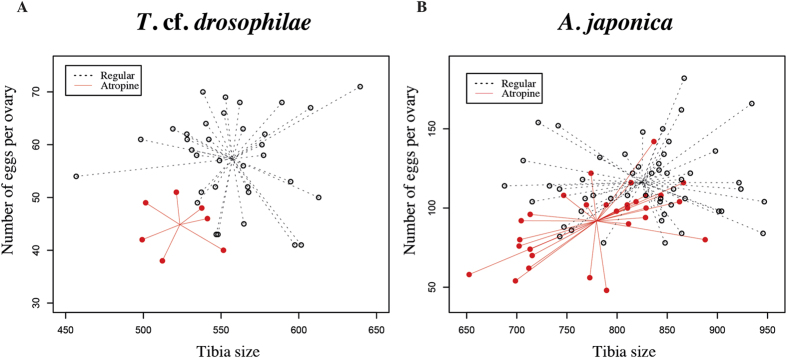
Impact of atropine in drosophila developmental medium on two life history traits of adult parasitoids. We recorded tibia size and number of eggs per ovary in (**A**) *T.* cf. *drosophilae*. (**B**) *A. japonica*. Broken black lines: parasitoids emerged from hosts reared on regular medium; solid red lines: parasitoids emerged from hosts reared on atropine medium.

Even after taking into account the decrease in size, parasitoids had a significantly lower number of eggs in their ovaries when emerging out of *D. suzukii* that fed on atropine medium. In *T.* cf. *drosophilae,* egg load decreased from 557.27 (SE = 5.98) to 523.49 (SE = 7.72) eggs per ovary (χ^2^_1_ = 15.49, *p-value* < 0.0001; Fig. 3A) and from 824.74 (SE = 8.9) to 779.43 (SE = 11.42) eggs per ovary (χ^2^_1_ = 67.05, *p-value* < 0.0001) in *A. japonica*. Finally, developmental time (corrected by the tibia length) was significantly increased for both parasitoids, *T.* cf. *drosophilae (average developmental time*: χ^2^_1_ = 0.0134, p-value < 0.003) and *A. japonica (average developmental time*: χ^2^_1_ = 0.0128, p-value = 0.045) when they emerged out of *D. suzukii* that were fed on atropine medium.

To verify that these results are not biased by a random effect of replicates (pseudo-replication), we used Generalized Linear Mixed Models to account for the difference in variance across replicates on the number of eggs per ovary and on the developmental time (see Methods). We observed that random variation across replicates does not affect our main conclusions on the effect of Drosophila developmental medium on parasitoid life-history traits (Number of eggs: Z-value = 3.848, p-value = 0.0001 for *T.* cf*. drosophilae*; Z-value = 7.690, p-value < 0.0001 for *A. japonica*. Average developmental time: t-value = 4.47, p-value < 0.0001 for *T.* cf*. drosophilae*; t-value = −2.27, p-value = 0.02 for *A. japonica*).

## Discussion

Our study shows that *Drosophila suzukii* uses atropine contained in its environment to perform trans-generational prophylactic medication and protect its progeny from parasitoids.

First, we show that eggs laid on atropine-containing medium do not protect *D. suzukii* from parasitization by its parasitoids (here, *Trichopria* cf. *drosophilae* or *Asobara japonica*) (Experiment 1). This experiment allows us to discriminate between two different behaviors, medication and anti-parasitization strategy. Anti-parasitization resistance strategy consists of laying eggs in a substrate containing components that are repellent for natural enemies^20^. For instance, ethanol in the development substrate of immature *Drosophila melanogaster* reduces the parasitization rate by parasitoid wasps^7^, especially by generalist parasitoids. We demonstrate that the parasitoids are not repelled by atropine and equivalently parasitize *D. suzukii* developing on atropine or regular medium.

Second, we show that *D. suzukii* is able to detect both the presence of parasitoids and the presence of atropine in its environment, resulting in higher rates of oviposition on atropine-containing substrates in the case of parasitoid presence (Experiment 2). The mechanism used by *D. suzukii* to detect parasitoids is still unknown. In *Drosophila melanogaster*^6^, the neuropeptide F is involved in the capacity of female flies to see wasps, and to switch their oviposition behavior to lay eggs in alcohol-laden food sources. Interestingly, the authors showed that *D. melanogaster* responded to the presence of larval parasitoids but not to pupal parasitoids. Here we show that *D. suzukii* is able to detect and modify its oviposition behavior in the presence of both pupal (*T.* cf. *drosophilae*) and larval parasitoids (*A. japonica*). Our result indicates that *D. suzukii* uses a medication strategy against parasitoids more broadly than *D. melanogaster*. In the presence of the pupal parasitoid *T.* cf. *drosophilae*, *D. suzukii* females lay significantly more eggs compared to conditions without parasitic pressure. This result suggests that, independently of the composition of the oviposition substrate, adult flies are also able to modify their behavior by increasing their oviposition frequency when parasitoids are detected in the developmental environment of their future progeny. A greater investment in egg production was already described in insects^21^, where immune challenged individuals compensate their shorter lifetime (due to enemies pressure) by ovipositing more eggs earlier in life. The main difference with our observation is that the lifetime expectation of adult females *D. suzukii* is not shortened by parasitoids when laying more eggs than usual. This observation opens new avenues to understand thoroughly the mechanisms of this protective behavior.

Third, results from experiments 3 and 4 highlight a positive effect of atropine on the development of parasitized *D. suzukii*, and a strong negative impact on several life history traits of these parasitoids. Many studies previously reported that host diet can modify the immune cell (*e.g.* hemocytes) concentration in insects^22^,^23^,^24^ and favor (or impact) the encapsulation reaction, *i.e.* an immune response leading to the neutralization of the foreign organism (*e.g.* parasitoid eggs or larvae). As we observed encapsulation reactions in flies resisting *A. japonica*, we could hypothesize that atropine or atropine-derived compounds act by up-regulating the quantity of hemocytes in *D. suzukii* larvae and increase their capacity to encapsulate parasitoids. Alternatively, atropine could elicit a direct negative toxicity effect on parasitoids, leading to the encapsulation of the dead parasitoid eggs. Note that observing encapsulation of *A. japonica* does not allow us to disentangle these two possible scenarios because direct effect of atropine on parasitoids, could later promote encapsulation of the inert foreign body (dead parasitoid egg or larvae). However, as encapsulation cannot occur during pupation^25^ and since we observe a tendency for higher resistance against the pupal parasitoid *T.* cf*. drosophilae*, we favor the scenario where atropine would have a direct toxic effect on parasitoids. The fact that *D. suzukii* probably faces higher difficulties in eliminating the dead *T.* cf*. drosophilae* parasitoid egg (because encapsulation cannot occur during pupation) may explain why resistance rates are lower than when *D. suzukii* is parasitized by the larval parasitoid *A. japonica*. Many herbivorous insect species are able to accumulate toxic defensive compounds (*e.g.* alkaloids) from their diet in their organism to protect against predators, parasites and parasitoids^26^,^27^,^28^,^29^,^30^. We suggest that immature parasitoids may be in contact with atropine present in the hemolymph or in the tissues of *D. suzukii*. The toxicity of atropine has been demonstrated to shape the dynamics of plant-insect coevolution^31^ and, under selective pressures, some insects evolved to tolerate this toxicity and to use plant properties to their own benefit and protection. Besides the case of *D. suzukii*, other insects are able to feed on plants of the *Atropa* genus^32^. For instance, when fed with *Atropa belladonna*, the larvae of the tobacco hornworm *Manduca sexta* excrete the plant alkaloids whilst the pupae of this Lepidoptera store atropine and its derived metabolites in their tissues^33^. Whether *D. suzukii* stores and detoxifies atropine at the larval and pupal stages is currently unknown and should be a target for future investigations. Furthermore, if this second hypothesis is true the evolution of the immune system of *D. suzukii* could be affected (see ref. 1). Indeed the direct effect of atropine storage on parasitoids provides an alternative to the costly immune response to parasitization.

Some previous studies on self and trans-generational medication in insects^5^,^6^,^34^,^35^ showed the existence of ecological trades-off: the substance used by the insect host to medicate had a detrimental effect on host survival in the absence of parasitoids. Here, we confirm the presence of such a trade-off, with a negative effect of atropine on the developmental time of *D. suzukii* in control conditions (without parasitoid pressure). This increased developmental time makes Drosophila immatures prone to attacks by parasitoids for a longer period of time. Furthermore, finding an adequate spot containing atropine to oviposit *in natura* can be challenging and should induce costs in terms of time and energy for *D. suzukii* adults.

Being a redoubtable pest, *D. suzukii* represents a serious threat for crop production across the globe^12^,^13^,^14^. Current strategies envisioned to reduce damages on crops involve the use of parasitoid species to control *D. suzukii*. Although some of these parasitoids efficiently reduce *D. suzukii* development in laboratory conditions^36^,^37^,^38^, our experiments suggest more variation in parasitoid virulence in natural environments where *D. suzukii* have access to toxins present in the local ecosystems. First, we show that when *D. suzukii* grows on atropine-containing medium, the mortality rate of both larval and pupal parasitoids is strongly increased. Second, our previous findings showed that *D. suzukii* is immunologically resistant against European larval parasitoids^38^ and, third, that many alkaloid-containing host plants are present *in natura* and used by *D. suzukii*^15^. All these parameters and particularly the composition of plant communities in the vicinity of crops have to be carefully considered before establishing any biological control strategies. Furthermore many scientists and agricultural companies suggest the use of natural enemies, *e.g.* parasitoids, as a biological tool to control *D. suzukii*^14^. As our study suggests that *D. suzukii* resists very virulent parasitoids, such control strategies may be inefficient in the long run and that extreme caution should be taken before possibly introducing new parasitoid species in Europe and the US (*e.g. Asobara japonica*).

So far, we do not know the origin of the ability of *D. suzukii* to use alkaloids as a weapon against its natural enemies. This strategy may be a legacy of co-evolutionary history in the indigenous area of the fly between *D. suzukii* and plants containing similar toxic compounds in their tissues. Indeed, many other wild and cultivated Solanaceae species are present in Japan^39^,^40^,^41^ and in the flora of China^42^, including endemic taxa. Even if *Atropa belladonna* is not naturally present in Japan (but introduced at least several decades ago), the other Asiatic Solanaceae taxa may host *D. suzukii* and have patterned the evolution of its tolerance to alkaloids.

Our study suggests, for the first time to our knowledge, that medication may contribute to promote an invasion success. The “Enemy Release Hypothesis”^10^ and “The Evolution of Increased Competitive Ability”^11^ state that alien species face a decreased pressure from natural enemies present in the introduced area, contributing to successful invasions. While *D. suzukii* benefits from the absence of parasitic pressure by specialized parasitoids in its new areas^6^,^38^, it also displays efficient physiological and behavioral (trans-generational medication) resistance reactions that further protect it against generalist enemies and may favor its expansion. For all these reasons, *D. suzukii* represents an extraordinary example of an invasive species that deserves full consideration in future studies of biological invasions.

## Methods

### Insect strains and rearing medium

The *Drosophila suzukii* strain used was initiated with 535 females collected in October 2011 in the Compiègne forest (latitude: 49°22′N, 2°54′E; 32–148 m altitude; 14417 ha; Picardie region; Northern France). Two parasitoid species known to be efficient against *D. suzukii* were tested^37^,^38^ (i) *Trichopria* cf. *drosophilae* (Hymenoptera: Diapriidae), a pupal parasitoid collected in 2012 in the Rhône valley, (France) and (ii) *Asobara japonica* Belokobylshij (Hymenoptera: Braconidae), a larval parasitoid collected in 2007 in Sendai (Japan) and offered by Professor J. J. M. Van Alphen (Leiden University, The Netherlands). Insects were mass-reared in the laboratory at 21 °C (+/−1 °C) under a LD 12:12 h photocycle and 70% relative humidity*. D. suzukii* flies were maintained in glass vials containing regular or atropine-laden media (see below). Parasitoids were maintained using a standard strain of *Drosophila melanogaster* originating from Sainte-Foy-Lès-Lyon, France (collected in 1994), and reared under the same laboratory conditions.

In our experiments, we reared *Drosophila suzukii* females on one of two possible media. Females grew on either (i) “regular medium” (as in ref. 37; Annex 1) or (ii) “atropine medium” (Annex 1), which was regular medium supplemented with 0.1% atropine (a0132 sigma; ≥99% −TLC- powder), the major alkaloid contained in the host plant *Atropa belladonna*. This percentage is in accordance with the observed concentration of atropine in the berries of *A. belladonna*^43^.

### Experimental design

Four experiments were conducted to test whether *D. suzukii* uses atropine to medicate against parasitoids. All experiments were conducted at 21 °C (climate chamber SANYO, type MLR-351H; temperature fluctuation: ±0.3 °C) with a 12:12 LD photoperiod and 70% relative humidity.

### Experiment 1: Parasitoid oviposition choice

The aim was to exclude a potential anti-parasitization resistance^7^,^20^, i.e. a substrate with repellent effect, in *D. suzukii*. We tested whether parasitoids were able to discriminate between the developmental medium of *D. suzukii,* and whether *D. suzukii* that were grown on atropine medium remained a potential target to parasitoid oviposition. This experiment was conducted with the two parasitoid species. We tested the ability of parasitoid females to discriminate between two *D. suzukii* pupae or larvae developed either on regular or on atropine medium by recording the oviposition choice of the parasitoid females. Two *D. suzukii* pupae (to test the choice of the pupal parasitoid *T.* cf. *drosophilae*) or two *D. suzukii* larvae (for the larval parasitoid *A. japonica)* were removed from their respective development glass vials along with 1 g of medium (regular or atropine medium) and placed at a distance of 2 cm from each other in a new vial (15 cm × 2 cm × 2 cm for *T.* cf. *drosophilae* or 3 cm × 0.5 cm × 0.5 cm for *A. japonica*). A drop of diluted brewers’ yeast was added on the top of each gram of medium to lure the larval parasitoid *A. japonica*. Then, one female parasitoid (five to eight days old and already mated) randomly chosen from the mass rearing was introduced in the vial. After its first oviposition, the choice was recorded and the female was removed. New pairs of *D. suzukii* pupae or larvae were used for each test. In total, 100 vials were monitored for *T.* cf. *drosophilae*, with twenty-five vials being monitored at the same time per day. A total of 101 choice-test were monitored for *A. japonica,* by batches of 5 simultaneous tests spread over two days (day 1: 63; day 2: 38 tests).

### Experiment 2: *Drosophila suzukii* oviposition choice in the presence of parasitoids

We aimed to test whether *D. suzukii* females were able to discriminate between a regular medium and an atropine medium and/or whether they modify their oviposition rate in the presence or absence of parasitoids. To check for a potential confounding effect of the rearing media on the subsequent laying choice made by drosophila females, flies were reared on either regular or atropine media before conducting the experiment. The two parasitoid species, *T.* cf. *drosophila* and *A. japonica,* were used. A factorial design was built with 6 modalities: 2 developmental media (regular or atropine; variable called *‘choice’*) and 3 parasitoid conditions (without parasitoid, with *T.* cf. *drosophilae* or with *A. japonica;* variable called *‘parasito’*). Fifty replicates were performed for each of the 6 modalities. Experiments were conducted over 10 days. Each day the 6 modalities were tested simultaneously 5 times (at the same time every day) between 10 am and 4 pm.

All drosophila and parasitoid adults used were five to eight days old and already mated. One drosophila female, one drosophila male and 3 parasitoid females of the same species were placed in a ventilated transparent plastic box (15 cm × 10 cm × 5 cm). In this box, we placed the two potential oviposition sites for the fly (dish of radius = 1 cm) separated by a distance of 5 cm from each other: one dish containing regular medium and the other one containing atropine medium. After 72 hours, the number of eggs laid by the fly was counted on each dish. The same protocol without the presence of parasitoids was used as a control.

### Experiment 3: Effect of atropine on the resistance of immature individuals of *D. suzukii* to parasitism, and on parasitoid virulence

We tested the effect of the developmental medium on the outcome of parasitization of *D. suzukii*. For each developmental medium (regular or atropine), we recorded the resistance of the flies against each parasitoid species, the emergence success of each parasitoid species, and the mortality of fly-parasitoid pairs.

In order to obtain parasitized *D. suzukii*, 5 drosophila immatures were placed in an empty vial (15 cm × 2 cm × 2 cm) and exposed to 10 female parasitoids. Pupae (5 days old at 21 °C) and second instar larvae (3 days old at 21 °C) were used for parasitization by the pupal parasitoids (*T.* cf. *drosophilae*) and by the larval parasitoids (*A. japonica*) respectively. We observed the parasitization and used only the parasitized immatures. Once a female parasitized an immature, we removed the parasitoid female and the parasitized host to avoid any pseudo replication due to several ovipositions of a given female or multi-parasitization by several parasitoid females. Parasitized immatures were then individually placed in micro-tubes (Durham; 7.25 cm × 0.50 cm) containing the same medium where they originally came from. The number of emerged drosophila allowed to estimate the successful drosophila resistance rate (number of emerged drosophila/number of parasitized drosophila). Parasitoids that successfully emerged from drosophila were counted to estimate the successful parasitoid development rate (number of emerged parasitoid/number of parasitized drosophila). In control groups, non-parasitized developing *D. suzukii* were individually placed in micro-tubes (Durham) containing regular or atropine medium to estimate natural mortality on the two media. The difference between the number of flies emerging from the control group (*i.e.* without parasitoid) and the total number of emerging insects (drosophila and parasitoids) after parasitization was used to estimate the mortality rate of the fly-parasitoid pairs following parasitization.

We used a factorial design with 6 modalities: 2 developmental media (regular or atropine; variable called ‘*DevD*’) and 3 parasitization states (non-parasitized, parasitized by *T.* cf. *drosophilae* or parasitized by *A. japonica;* variable called ‘*parasito*). One hundred replicates were performed for each of the six modalities. Experiments were conducted over 20 days. Each day the 6 modalities were tested simultaneously 5 times (at the same time every day). Both fly and parasitoid emergences were observed and counted every day (twice a day, at 9 am and 7 pm), during the 45 days following the parasitization.

### Experiment 4: Effects of atropine on parasitoid life-history traits

All parasitoid females that successfully emerged in Experiment 3 (from flies grown either on regular or atropine medium) were used to measure the effects of atropine on three relevant proxies of insect fitness: development time, tibia size and fecundity^44^,^45^.The development time was measured as the time elapsed between the parasitoid parasitization and the emergence of an adult parasitoid. Emerging parasitoid females were immediately and individually isolated in a vial containing food (moist cotton and honey) to allow the maturation of eggs in ovaries. Five days later, tibia size and potential fecundity, estimated as the total number of eggs in one ovary, of each individual were measured. An Axiovision imaging software (Carl Zeiss Axiovision Release 4.8.1) was used to count the eggs and to measure tibia sizes on the collected images. Measures were performed on 34 females of *T.* cf. *drosophilae* and 51 females of *A. japonica* emerged from drosophila reared on regular medium, and on 7 females of *T.* cf. *drosophilae* and 29 females of *A. japonica* emerged from drosophila reared on atropine medium.

### Statistical analysis

As the two parasitoid species have very different characteristics and life cycles (one larval and one pupal parasitoid), they were considered separately in analyses. In experiment 2, to identify the factors that significantly influenced the number of eggs laid by *D. suzukii*, a generalized linear model (GLM), with a Poisson error and log link was performed. We used the medium chosen by the females to oviposit (variable called ‘*choice*’ and including 2 modalities: regular or atropine medium), the rearing medium the females originated from (called ‘*devR’* and also including 2 modalities: regular or atropine medium), presence/absence of parasitoids of a given species (factor called ‘*parasito’*) and the replicate (factor called ‘set’) as explanatory variables.

In experiment 3, we used Wilcoxon rank sum test (with continuity correction) to analyze the effect of atropine on *D. suzukii* developmental time. We used generalized linear models (Poisson error and log link) to examine the drivers of the number of drosophila emerging after parasitization, the number of emerging parasitoids and the number of dead fly-parasitoid pairs. For the number of emerging drosophila, the explanatory variables were the drosophila developmental medium type (‘*devD’* with 2 modalities: regular and atropine), the presence/absence of parasitoids (‘*parasito’*) and the replicate (‘set’). For the GLMs on the number of parasitoids emerging from parasitized hosts and the number of dead fly-parasitoid pairs, the explanatory variable used were the drosophila developmental medium type (‘*devD*’ with 2 modalities: regular and atropine) and the replicate (‘set’).

In experiment 4, because of the classic correlation between tibia size (a proxy of adult size) and the development time and egg load per ovary, we corrected these two life traits by the tibia size, by introducing it as the first explanatory variable in generalized linear models (GLMs) (type I analysis in R software, as in ref. 46). The other explanatory variable was developmental medium. Egg-load and developmental time were analyzed with Poisson error and Gamma error with inverse link for egg-load and development time, respectively. The tibia size was analyzed by a linear model because of the unbalanced design (only 7 samples were available for traits in the parasitoid *T*. cf. *drosophilae* emerged from *D. suzukii* reared on atropine medium). We performed permutation tests to assess the robustness of our statistical conclusions (see supplement for a detailed description, Annex 3). All the interactions between explanatory variables were included in the models. Models with several explanatory variables were simplified by a backward procedure with a likelihood ratio test. As parasitoids used in Experiment 4 were those emerging in Experiment 3, statistical analyses using generalized linear models described above could be biased by pseudo-replication effects: if some replicates (sets) in Experiment 3 generated much more parasitoids than other replicates, this could drive the difference in parasitoid life-history traits between control and atropine developmental conditions. To control for the random effect of replicates, we used Generalized Linear Mixed Models and considered the replicates as a variable with a random effect on the response variable (either the number of parasitoid eggs or the parasitoid developmental time) and the Drosophila developmental medium as a variable with fixed effects. We used the ‘glmm’ function with a Poisson distribution from the ‘glmm’ R package for the number of parasitoid eggs, and the ‘glmer’ function with a Gamma distribution from the ‘lme4’ R package for the developmental time.

All statistical analyses were performed with the R freeware statistical package (R Development Core Team 2013, version 3.2.1).

## Additional Information

**How to cite this article:** Poyet, M. *et al*. The invasive pest *Drosophila suzukii* uses trans-generational medication to resist parasitoid attack. *Sci. Rep.* **7**, 43696; doi: 10.1038/srep43696 (2017).

**Publisher's note:** Springer Nature remains neutral with regard to jurisdictional claims in published maps and institutional affiliations.

## Supplementary Material

Supplementary Information
