# Supplementary material for: The invasive pest Drosophila suzukii uses trans-generational medication to resist parasitoid attack
Source: Sci Rep. 2017 Mar 13;7:43696. doi: 10.1038/srep43696 (PMC5347128; doi:10.1038/srep43696)
Supplement: Supplementary Information [file srep43696-s1.pdf]

The invasive pest *Drosophila suzukii* uses trans-generational medication to resist parasitoid attack

Poyet M, Eslin P, Chabrerie O, Prud'hommes S M, Desouhant E & Gibert P

Supplementary Information

<u>Annex 1: Media recipes</u>

Standard medium recipe (i.e. banana medium):

Mix and heat 20g agar in 500mL H2O, then add 400 g of mashed banana, 50g of brewers' yeast, 30g of flour, 20g of sugar. Let cool below 30C° before adding 4g of nipagine previously diluted in 30mL of alcohol 70°. Finally add 550mL of H2O. Mix all ingredients for 5min.

Atropine medium:

Follow the same recipe as for regular medium except for the atropine (0,1%) that has to be diluted in 30mL of alcohol 70° with the nipagine.

Annex 2:

Mean (± S.E.) developmental time (in days) of *Drosophila suzukii* from eggs to adults in regular medium and in atropine containing medium.

	Regular medium	Atropine medium
Control	16.11± 0.20	17.56 ± 0.26
After infestation by *A. japonica*	16.76 ± 0.28	17.84 ± 0.21
After infestation by *T.* cf. *drosophilae*	16.60 ± 0.24	17.08 ± 0.34

Annex 3:

Permutation tests and impact of atropine on life-history traits of parasitoids.

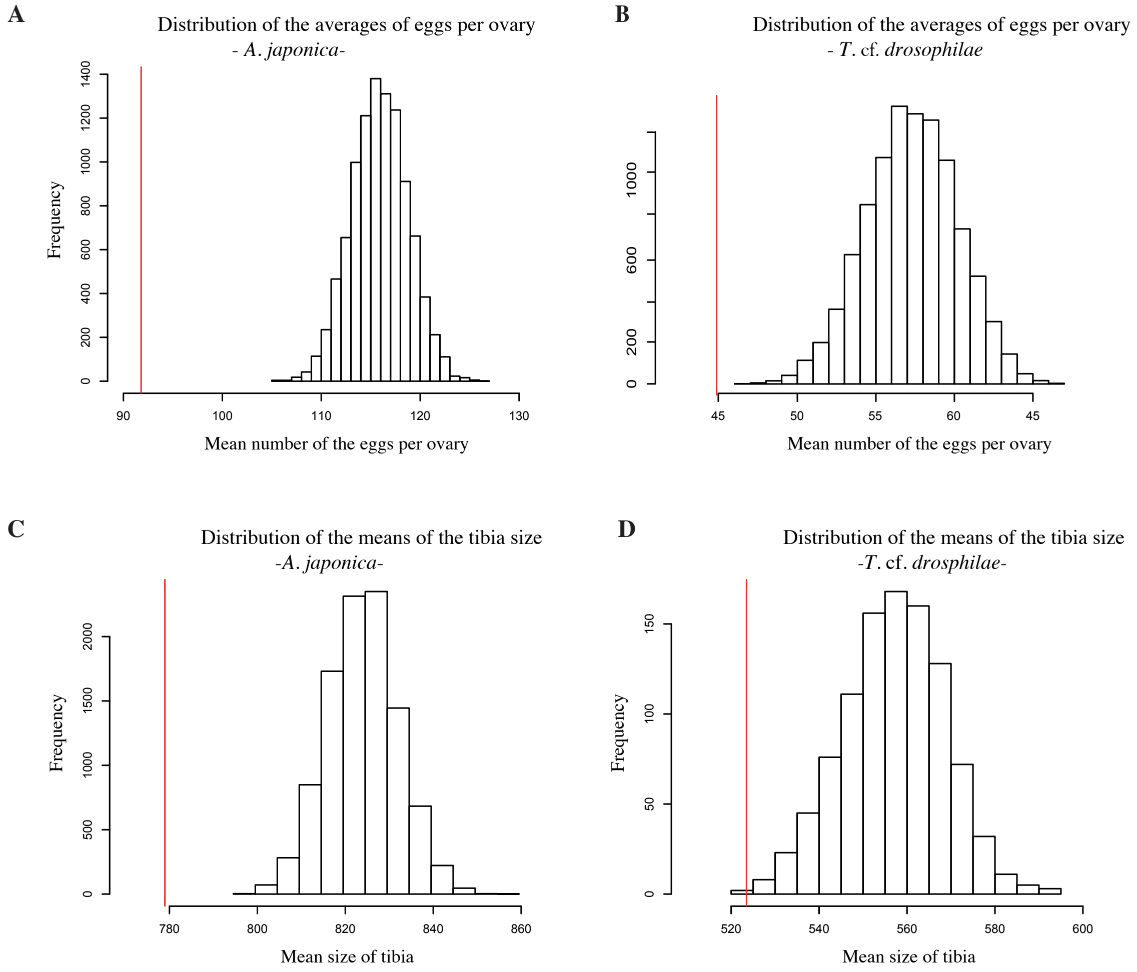

A: The histogram represents the distribution of the mean number of eggs per ovary for 10,000 random draws of 34 out of 55 *A. japonica* individuals emerged from *D. suzukii* reared on regular medium. In our experiment, 55 and 34 *A. japonica* individuals emerged from *D. suzukii* reared on regular and atropine medium, respectively. The red line shows the mean of eggs per ovary for these 34 individuals. **B**: Same as in A, for *T.* cf. *drosophilae*. In our experiment, 29 and 7 *T.* cf. *drosophilae* individuals emerged from *D. suzukii* reared on regular and atropine medium, respectively. **C**: Same as in A, for the impact of atropine on the tibia size of *A. japonica*. **D**: Same as in B, for the impact of atropine on the tibia size of *T.* cf. *drosophilae*.
